# Bridging Convolutional Neural Networks and Transformers for Efficient Crack Detection in Concrete Building Structures

**DOI:** 10.3390/s24134257

**Published:** 2024-06-30

**Authors:** Dhirendra Prasad Yadav, Bhisham Sharma, Shivank Chauhan, Imed Ben Dhaou

**Affiliations:** 1 Department of Computer Engineering & Applications, G.L.A. University, Mathura 281406, India; dhirendra.yadav@gla.ac.in (D.P.Y.); shivank.chauhan@gla.ac.in (S.C.); 2 Centre of Research Impact and Outcome, Chitkara University, Rajpura 140401, Punjab, India; 3 Department of Computer Science, Hekma School of Engineering, Computing, and Design, Dar Al-Hekma University, Jeddah 22246-4872, Saudi Arabia; 4 Department of Computing, University of Turku, 20014 Turku, Finland; 5 Higher Institute of Computer Sciences and Mathematics, Department of Technology, University of Monastir, Monastir 5000, Tunisia

**Keywords:** building crack, CNN, transformer, attention, classification

## Abstract

Detecting cracks in building structures is an essential practice that ensures safety, promotes longevity, and maintains the economic value of the built environment. In the past, machine learning (ML) and deep learning (DL) techniques have been used to enhance classification accuracy. However, the conventional CNN (convolutional neural network) methods incur high computational costs owing to their extensive number of trainable parameters and tend to extract only high-dimensional shallow features that may not comprehensively represent crack characteristics. We proposed a novel convolution and composite attention transformer network (CCTNet) model to address these issues. CCTNet enhances crack identification by processing more input pixels and combining convolution channel attention with window-based self-attention mechanisms. This dual approach aims to leverage the localized feature extraction capabilities of CNNs with the global contextual understanding afforded by self-attention mechanisms. Additionally, we applied an improved cross-attention module within CCTNet to increase the interaction and integration of features across adjacent windows. The performance of CCTNet on the Historical Building Crack2019, SDTNET2018, and proposed DS3 has a precision of 98.60%, 98.93%, and 99.33%, respectively. Furthermore, the training validation loss of the proposed model is close to zero. In addition, the AUC (area under the curve) is 0.99 and 0.98 for the Historical Building Crack2019 and SDTNET2018, respectively. CCTNet not only outperforms existing methodologies but also sets a new standard for the accurate, efficient, and reliable detection of cracks in building structures.

## 1. Introduction

In recent decades, the emphasis on preserving historic structures and understanding civil infrastructure safety has amplified the focus on structural damage assessment. Observable defects in building exteriors, like fractures, serve as essential indicators of structural integrity [1,2]. Traditionally, building conditions are inspected by sending workers to inspect for cracks. This conventional method has limitations, as its reliability heavily depends on the expertise of the professionals. The anticipated compound annual growth rate for the global structural health monitoring market is 17.9% and is estimated at USD 1.5 billion in 2018 and projected to be USD 3.4 billion in 2023 [3]. Civil infrastructures are the foundation of a nation’s economy and the vital core of prosperous communities. After a few years, these structures face different forms of deterioration, and due to this, the strength of buildings decreases [4]. In concrete structures, cracks can be caused by environmental exposure, aging, recurrent loading, and surpassing load limitations allowed [5]. Classical methods like a flashlight or magnifying glass can be used to detect any flaws. In addition, a tapping test can be used to detect cracks. In this technique, a small hammer is used to tap the location of the crack. An echoing sound can suggest the presence of a space or separation below the surface. Furthermore, dyes can be inserted into the crack region to determine the depth of the crack. 

Recently, image processing techniques have been used to automate the crack inspection process. In this technique, images are captured using cameras from the suspected region. After that, texture and shape features are extracted from the images to train the machine learning models. Over the last decade, the utilization of deep learning methods has grown in construction management. Deep learning techniques have successfully monitored construction activity, including equipment use and resource management [6,7]. Several methods investigated building cracks using traditional image processing algorithms, like finding the edges of the crack region [8,9], regular angle evaluations to reconstruct the 3D models [10,11], pixel intensity variations [12,13], and finding center line detection algorithms [14,15]. According to studies, deep learning approaches are more robust to imaging fluctuations [16]. Recently, ViT (vision transformer)-based methods have been widely used in the image classification task. However, they require large training samples for higher classification accuracy. 

Several machine learning and deep learning methods have been utilized to solve the challenges imposed by complex building structure crack identification. However, machine learning methods require hand-crafted features for crack classification. In contrast, deep learning improved performance by extracting shallow high-dimension features. Moreover, ViT-based methods require large training samples. Nevertheless, the lack of a global co-relation of features in CNNs makes them less reliable. We proposed a convolutional and ViT (vision transformer)-based CCTNet for robust and reliable identification of the concrete defect with small training samples. Our CCTNet improved the spatial features of the crack images through window-based self-attention and local attention modules. Furthermore, we evaluated our model’s performance on three datasets and compared them with the classical and ViT methods. 

The main contribution of the proposed method can be summarized as follows:

(a) The proposed model enhanced the crack identification accuracy through the utilization of an increased number of input pixels compared to the conventional approach, resulting in a more precise detection level.
(b) The co-relation of the local and global spatial features is improved through convolution channel attention with window-based self-attention. The CNN localized feature extraction and self-attention mechanisms’ contextual considerations make this fusion technique more effective in describing crack patterns.
(c) We designed an overlapping cross-attention module to enhance the interaction of features across adjacent image windows. This makes CCTNet more coherent for the inspection of the visual data, which is important for identifying complex cracks.
(d) We validated the superiority of CCTNet on the Historical Building Crack2019, SDTNET2018, and DS3. DS3 is created by our team, and a detailed description of DS3 is provided in Section 4.1.

The rest of the manuscript is organized as follows. In Section 2, we have provided a detailed review of the past methods. Section 3 describes the proposed CCTNet architecture. In Section 4, the datasets and quantitative results have been discussed. Further, in Section 5, a discussion of the performance measures has been elaborated. Finally, in Section 6, the conclusion and future scope of the proposed method have been added.

## 2. Related Work

In the past, several machine learning and deep learning methods have been developed for building crack detection. Le et al. [17] set an FBG (Fiber Bragg Grating) accelerometer to find low-frequency excitation using an aluminum mass block. The FBG acceleration sensor resonant frequency is close to 143 Hz, and the FBG accelerometer sensitivity signal ranges from 6.74 pm/g to 26.64 pm/g. Yu et al. [18] designed a robot to find bridge cracks. The robot has sensors and a microcontroller that help to avoid obstacles. It contains a charge-coupled device (CCD) camera that identifies damages from the complex location on the bridge and provides length, width, and position. Yuan et al. [19] developed the utilized ResNet50 as a backbone for building crack detection. In addition, channel attention is utilized to enhance the feature and achieved 83.07% IOU (intersection over union) value. Kim et al. [20] developed a deep learning method to visually examine surface cracks on buildings. The authors suggested a shallow architecture based on a CNN for constructing a crack-detecting system. Alipour et al. [21] employed ML algorithms, specifically random forest, and decision trees, to determine the load capacity of massive bridges. The model was trained using a dataset of more than 40,000 structures of national concrete slab bridges.

Chen et al. [22] constructed a crack detection system using a transfer learning based CNN approach and obtained 94% classification accuracy. Furthermore, Snow et al. [23] employ neural networks (NNs) and CNNs to identify the flaws in the layer-wise buildings in images. They train the models on single-layer and multilayer images of various lighting conditions. CNNs were more generalized compared to NNs. Alipour and Harris et al. [24] designed a residual CNN-based model to investigate the model performance on separate asphalt and concrete cracks. The training and validation were conducted on the cross-dataset to demonstrate the effectiveness of the model on different materials. Zeng et al. [25] identified pixel-level cracks in a building using a FPAFFN (Feature Fusion Network). To enhance the spatial features, an attention mechanism was applied, and model performance was evaluated on the Cracktree200 and CRACK500 datasets. In similar research, Torok et al. [26] reconstructed 3D images to identify cracks. The 3D model, based on pictures, is capable of identifying and examining damage in three dimensions. It also showcases the robotic platform employed to capture the series of images necessary for the reconstruction, thereby minimizing potential risks. In addition, the 3D crack detection method can utilize 3D mesh models as sources for data collection.

Paulo et al. [27] designed a software tool building management system (BdMS) for building inspection to characterize and quantify several facade anomalies. First, the BdMS collects the building data visually and photographically and then performs accurate and quick defect detection and measurements on building facades. Pereira and Pereira et al. [28] use image processing algorithms to detect cracks in building facades. The algorithm should be installed in an Unmanned Aerial Vehicle (UAV). They select two image processing algorithms for classification and crack detection. Further, a UAV is applied for image acquisition compared to the baseline, with implementation running on an embedded processor. Kersten et al. [29] identified the building health by unmanned aircraft systems (UASs), and automated image analysis was determined and evaluated. Furthermore, they automatically identified affected areas using a method for extracting 3D object information.

Hoskere et al. [30] developed an algorithm for automatic crack identification using each pixel-wise image through a deep CNN. The output of the six types of cracks in the resulting image is segmented and represents the damaged portion outlined. Guo et al. [31] utilized a deep learning algorithm to find facade defect classification. They proposed a layer-based characterization rule for facade defects. Further, they compared the previous method classification accuracy CNN and meta-learning-based CNN model on the imbalance dataset. The time series-based-methods have been shown to be effective tools for automatic structure health monitoring of civil infrastructures [32,33]. 

In short summary, ML-based methods showed less performance due to hand-crafted features. The classical CNN methods improved performance but lack the global correlation of the spatial features. Moreover, ViT-based methods utilized the global correlation of the features. However, they require large training samples for the training of the model [34].

## 3. Proposed Method

**Figure 1:**
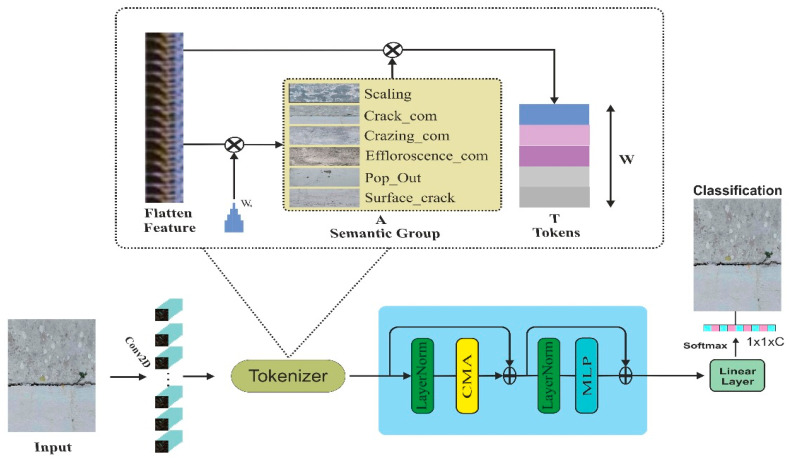
The proposed CCTNet architecture for crack detection.

We have investigated recent methods for identifying cracks in buildings. Traditional pre-trained deep CNN models such as VGG16, Inception V4, GoogleNet, ResNet50, and AlexNet showed suboptimal performance due to a mismatch between the classes in the ImageNet dataset. Some studies first trained the deep learning models using specifically chosen datasets. Afterwards, the model undergoes validation on the dataset, demonstrating exceptional performance. Nevertheless, the computational costs associated with these methods are significantly elevated due to the many trainable parameters. Furthermore, these techniques only extract shallows spatial features. Recently, ViT-based methods showed high performance in image classification through local and global attention to the features. However, they require a large training sample and costs. In the proposed method, a convolution and composite attention transformer (CCAT) effectively employs more input pixels to identify cracks. It combines convolution channel attention and window-based self-attention to leverage the benefits of both methods. In addition, we incorporate an overlapping cross-attention module to enhance the synergy between adjacent window features and consolidate the collective information across windows. The detailed architecture of the suggested approach is shown in Figure 1.

### 3.1. The Vision Transformer (ViT)

The vision transformer (ViT) is a deep learning framework that applies the transformer framework and is initially used in natural language processing in computer vision. The system transforms input images into patches, encodes relevant information, and utilizes stacked transformer blocks to preserve spatial associations between features. The ViT has demonstrated its proficiency in image classification, object identification, and segmentation [35,36]. The typical transformer architecture achieves long-range input dependencies by incorporating the multi-head self-attention layer (MHA) [37]. The Layer Normalization (LN) and Feed-Forward Network (FFN) are the fundamental components of the transformer model. Based on the feature $x\in R^{m\times c}$, the operation of the ViT is defined as follows:

(1) $$x_{i}^{m}=LN(x_{i})$$

(2) $$x_{i}^{\prime }=MHA(x_{i}^{m},x_{i}^{m},x_{i}^{m})+x_{i}$$

(3) $$MHA(Q,K,V)=concat(H_{1},H_{2},\ldots \ldots H_{m})W^{o}$$

(4) $$H_{j}=Attention(QW_{j}^{q},KW_{j}^{k},VW_{j}^{v})$$

(5) $$Attention(q,k,v)=soft\max\left(\frac{qk^{T}}{\sqrt{d}}\right)v$$

(6) $$x_{i+1}=FFN(LN(x_{i}^{\prime }))+x_{i}^{\prime }$$

where d is the dimension head and $W^{o},W_{j}^{q},W_{j}^{k},W_{j}^{v}$ are the weight matrices; with an increase in the token size, computation costs quadratically increase. Therefore, in this study, we develop a convolution and composite attention transformer (CCAT) that reduces the costs and improves classification accuracy.

### 3.2. The Convolution and Composite Attention Transformer (CCAT)

**Figure 2:**
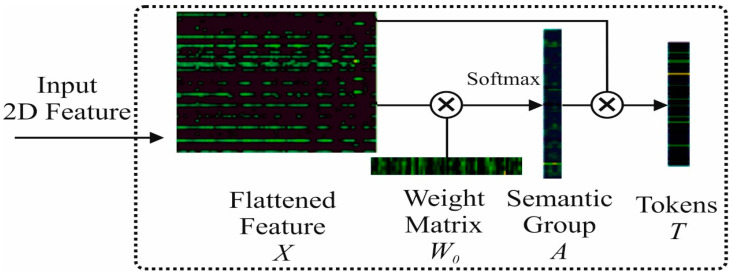
The tokenization process of the proposed method.

In the proposed study, we first extracted the spatial features using a 2D convolution layer with a kernel size of 64. After that, tokens are generated by flattening the features and applying the Xavier standard norm. The input weight $W_{b}\in R^{64\times 4}$ is multiplied by the initial weight matrix that produces semantic group $A\in R^{81\times 4}$. Finally, we multiplied the transpose of A with feature vector Z to generate token $T\in R^{4\times 64}$. The tokenization process is shown in Figure 2.

In the composite attention transformer block, the operation on the feature Z in the local windows is defined as follows:

(7) $$\begin{array}{l} Z_{N}=LN(Z),\\ Z_{M}=(s)W-MSA(Z_{N})+\beta CAM(Z_{N})+Z\\ Z^{\prime }=MLP(LN(Z_{M}))+Z_{M} \end{array}$$

**Figure 3:**
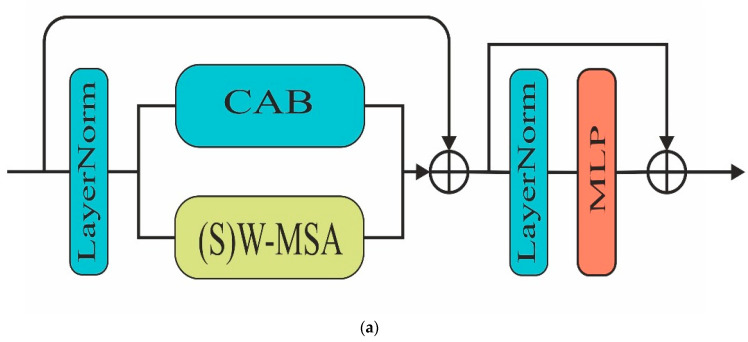

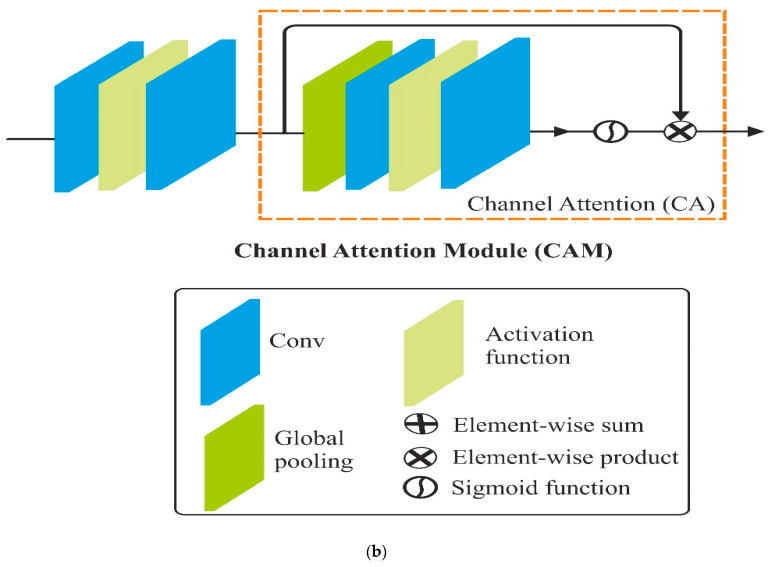
The composite multi-head attention block and channel attention block are shown in (**a**,**b**), respectively.

Further, we calculated the Q (query), K (key), and V (value) from the feature $Z\in R^{P^{2}\times c}$ as follows:

(8) $$Q=ZW_{Q},K=ZW_{K},V=ZW_{V}$$

where $W_{Q}$, $W_{K}$, and $W_{V}$ are the projection matrix shared among the different local attention windows. Finally, the attention on the local window is calculated as follows:

(9) $$Attention(Q,K,V)=Soft\max(QK^{T}/\sqrt{d}+\eta )V$$

where η = positional encoding bias to the adjust learnable parameters. The CMA (composite multi-head attention) and CAB (channel attention block) are shown in Figure 3. The convolution channel attention block improves the focus of the model on the relevant parts of the image and adjusts the weight of different channels dynamically. As we can see in Figure 3b, it has three major components: a convolution block, global average pooling, and an activation function. First, we applied a convolution block to generate high-dimensional spatial features. After that, a global average pooling operation is performed to create a feature vector of each channel. Furthermore, we pass the feature vector through another fully connected layer. Finally, the sigmoid activation function is applied to generate the final attention score. As shown in Figure 3a, a window-based multi-head self-attention block has been implemented in parallel with channel attention. The W-MSA reduces the computation costs of the attention mechanism in an input image with long sequence embedding through computation in a local window. In this technique, we first divided the input into fixed-sized non-overlapping patches. After that, self-attention is calculated on each window. Finally, we merged each local window’s attention to generate an enhanced attention map.

The overlapping cross-window attention mechanism is implemented in the W-MSA block. In W-MSA, attention is calculated locally in each window, and for adjacent widows, merging of attention is performed using overlapping cross-window attention. The pseudocode of the overlapping cross-window attention techniques is as follows:
def OCA (Input_FM, WS, OS): # Step 1: Partition input into overlapping windows windows = PO (Input_FM, WS, OS)   # Step 2: Apply cross-attention within each overlapping window for each window in windows:  queries, keys, values = extract_qkv (window)  attention_output = cross_attention (queries, keys, values)  STO (attention_output, window.position # Step 3: Merge overlapping windows output_feature_map = MOW (attention_outputs, OS) return output_feature_map def PO (Input_FM, WS, OS): windows = [] step_size = WS − OS height, width, channels = dimensions (Input_FM)  for i in range (0, height − WS + 1, step_size):  for j in range (0, width − WS + 1, step_size):   window = Input_FM[i:i+WS, j:j+WS,:]   windows.append (window) return windows def extract_qkv (window): # Extract queries, keys, values from the window queries = linear_projection (window) keys = linear_projection (window) values = linear_projection (window) return queries, keys, values def cross_attention (queries, keys, values): # Compute attention scores scores = softmax(matmul (queries, transpose(keys))/sqrt(dim(keys))) attention_output = matmul (scores, values) return attention_output def STO(attention_output, position): # Store the attention output in a buffer for merging buffer[position] = attention_output def MOW(attention_outputs, OS): output_feature_map = initialize_output_feature_map() count_map = initialize_count_map() # To count the number of contributions to each position for position, attention_output in attention_outputs:  for i in range(WS):   for j in range(WS):    output_feature_map[position[0] + i, position[1] + j,:] += attention_output[i, j,:]    count_map[position[0] + i, position[1] + j] += 1  # Average the overlapping regions for i in range(height (output_feature_map)):  for j in range (width(output_feature_map)):   if count_map[i, j] > 0:    output_feature_map[i, j,:] /= count_map[i, j] return output_feature_map

The features extracted from the composite attention transformer are passed to the SoftMax layer to classify the different building cracks. The SoftMax function converts the logits into the probability values as follows:

(10) $$P(y=L|F^{\left(i\right)})=\frac{e^{F^{\left(i\right)}}}{\sum_{L=0}^{N}e_{N}^{F^{\left(i\right)}}}$$

where $w_{0}y_{0}$ is a bias value added in each iteration to classify cracks, and $F=w_{0}y_{0}+w_{1}y_{1}+w_{2}y_{2}\ldots +w_{N}y_{N}$. The loss is an essential factor in evaluating the model’s performance. The high loss for the training and validation shows that the model is less reliable. We have applied binary cross-entropy and categorical cross-entropy functions to calculate the loss of binary and multi-class classification. The algorithm of the proposed methods is as follows (Algorithm 1).
**Algorithm 1.** The Proposed Method for Structure Detection Categorization(1) Resize the input image to 300 × 300 × 3 pixels (2) Apply dataset augmentation technique to increase the size of the DS3 (3) For i = 1 to 200 do,    (a) Extract spatial features through a convolution block    (b) Generated tokens $T\in R^{64\times 4}$ on the flattened features    (c) Generate $Q=ZW_{Q},K=ZW_{K},V=ZW_{V}$  End (4) Plot the confusion matrix of the datasets (5) Plot the training and validation loss curve (6) Plot precision-recall curve

## 4. The Experimental Results

In this section, we have discussed the experimental results of CCTNet on the Historical Building Crack2019, SDTNET2018, and proposed DS3.

### 4.1. Datasets

The Historical Building Crack2019 (https://data.mendeley.com/datasets/xfk99kpmj9/1, accessed on: 20 October 2023) dataset has approximately 3886 carefully compiled images, exhibiting the concrete surfaces from historic buildings. This collection has over 40 authentic images from a historic mosque (Masjid) in Cairo, Egypt. The dataset comprises 757 images depicting cracked surfaces and 3139 images showing non-cracked surfaces. The photographs were taken with a Canon EOS REBEL T3i digital camera with a 5184 × 3456 pixel resolution. The shots were captured over two years, particularly between 2018 and 2019. Each image in the dataset has been meticulously annotated and is designed to facilitate the training and validation of automated methods for detecting cracks, assessing crack severity, and segmenting cracks [38].

SDNET2018 (https://digitalcommons.usu.edu/all_datasets/48/, accessed on: 12 November 2023) is a collection of annotated images that aims to assist in the training and validating of artificial intelligence-based algorithms for detecting cracks in concrete. The dataset contains 560,000 images of cracks and non-cracks observed on the concrete surfaces on walls, bridges, and pavement. All the images were captured using a 16-megapixel Nikon camera, maintaining a fixed distance of 500 mm without utilizing any zoom functionality. The images in the dataset have a 4068 × 3456 pixel size with a crack range of 0.06 mm to 25 mm. The complete image was carefully partitioned into smaller sub-images measuring 256 × 256 pixels each to facilitate analysis. Each sub-picture corresponds to a physical region measuring around 60 mm × 60 mm, whereas the original full image reflects an actual area of approximately 1000 mm × 850 mm. Furthermore, the collection includes images that portray various challenging situations, such as shadows, differences in surface texture, changes in size, edges, holes, and debris in the backdrop [39].

**Figure 4:**
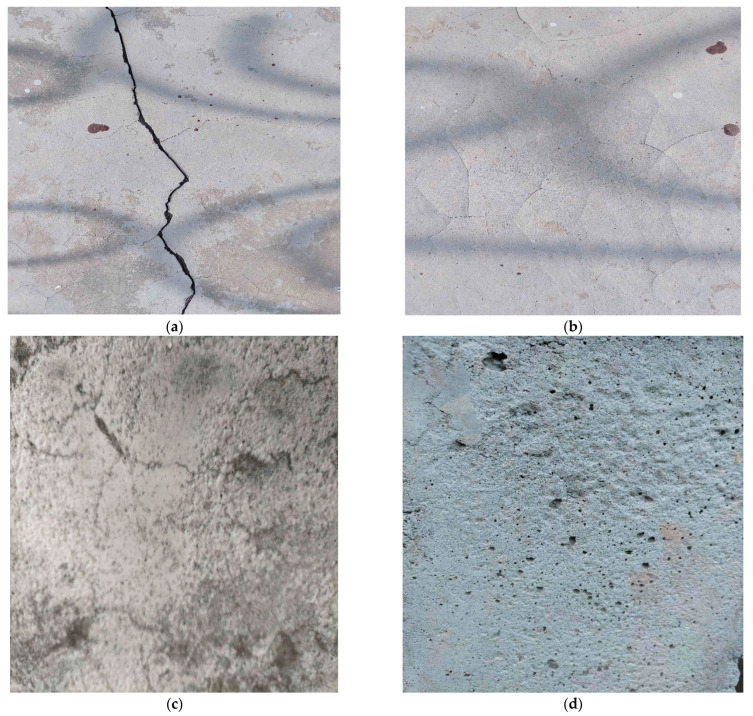

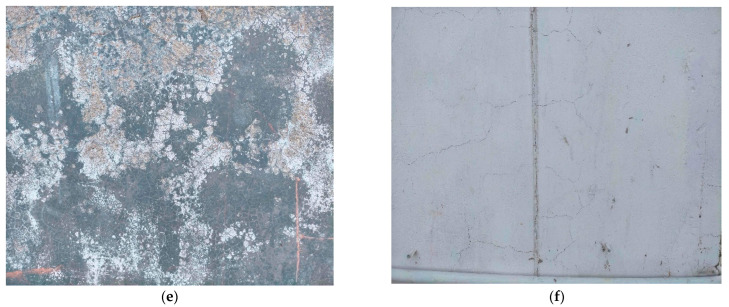
Sample images of the Crk, Crz, Efs, Pot, Scl, and Sfc are shown in (**a**–**f**), respectively.

The third dataset (DS3) was generated by capturing images of the concrete wall surface with an Android phone. The Android phone has a dual-lens camera with a wide-angle capability and a high resolution of 200 megapixels. We have successfully captured the existence of Cracks (Crk), Crazing (Crz), Efflorescence (Efs), Pop-out (Pot), Scaling (Scl), and Surface Cracking (Sfc). Figure 4 displays the sample images. After the initial collection of 2155 raw pictures, 2115 images were selected for subsequent processing. This was achieved by excluding pictures not meeting the stated quality criteria. Data augmentation methods, including rotation, horizontal flip, vertical flip, and shear, enlarged the size of the dataset. This was important due to the small size of the DS3 dataset. After completing the augmentation process, every class possesses 2000 images. Table 1 shows the specification of the image-capturing device for DS3. Additionally, each image was stored in the .jpg file format, with a resolution of 11,555 pixels in width and 8655 pixels in height. Figure 4 displays sample images of DS3.

### 4.2. Experimental Settings

We experimented with the proposed model on a Windows 10 operating system using Python 3.9. The computational hardware consisted of an NVIDIA Quadro TRX4000 GPU, complemented by 128 GB of RAM and two graphics cards, each featuring 8 GB of memory. Before being inputted into CCTNet, the images were resized to a resolution of 300 × 300 pixels. Subsequently, the model underwent training in batches of 64 for every iteration. The learning rate was initially set to 0.0001 to enhance the training process progressively. We allowed the model to learn and adjust its parameters over 200 epochs.

### 4.3. Performance Evaluation on the Historical Building Crack2019 Dataset

**Figure 5:**
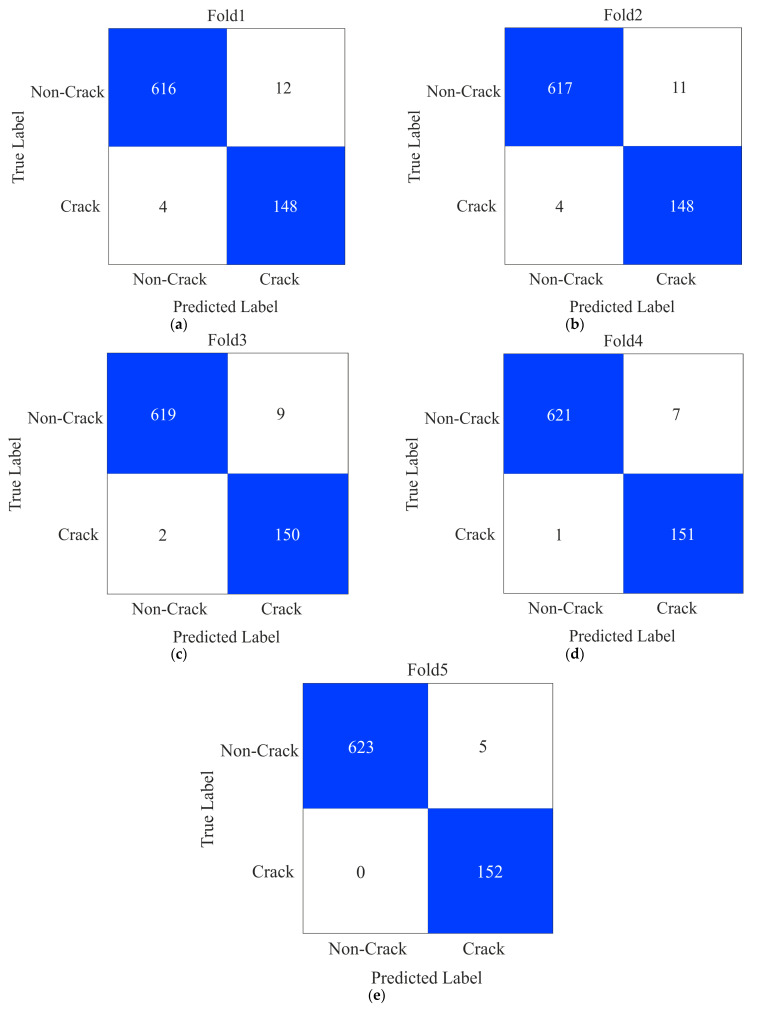
Confusion matrices of CCTNet on the Historical Building Crack2019 dataset (**a**) Fold1 (**b**) Fold2 (**c**) Fold3 (**d**) Fold4 and (**e**) Fold5.

The Historical Building Crack2019 dataset has 757 images depicting cracked surfaces and 3139 photographs depicting non-cracked surfaces. In order to mitigate bias performance, we investigated the model’s efficacy via a five-fold cross-validation technique. In a five-fold cross-validation strategy, the dataset is partitioned into five subsets, with four subsets used for training and one subset used for validation. Initially, we adjusted the dimensions of the image to a precise size of 300 × 300 pixels. Subsequently, the data were inputted into CCTNet for training, utilizing a batch size of 64 for 200 epochs. The Adam optimizer expedited the training process by utilizing an initial learning rate of 0.0001. The confusion matrix for each fold is depicted in Figure 5. We can notice in Fold1 that the FP (false positive) value is 12 and the FN (false negative) value is 4. In substituent folds, it decreases. Finally, in Fold 5, CCTNet has five FP and zero FN values.

From the confusion matrices shown in Figure 5, we calculated kappa, recall, precision, F1-score, and accuracy of CCTNet on the Historical Building Crack2019 dataset, as shown in Table 2. In Table 2, we can notice that in Fold_1, the kappa value is 93.64%, and it gradually increases in Fold_2, Fold_3, Fold_4, and Fold_5. Similar trends can be observed for the other performance measures.

### 4.4. Performance Evaluation on the SDTNET2018 Dataset

**Figure 6:**
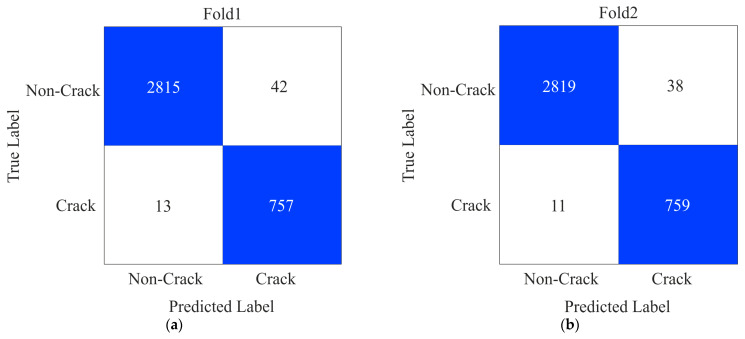

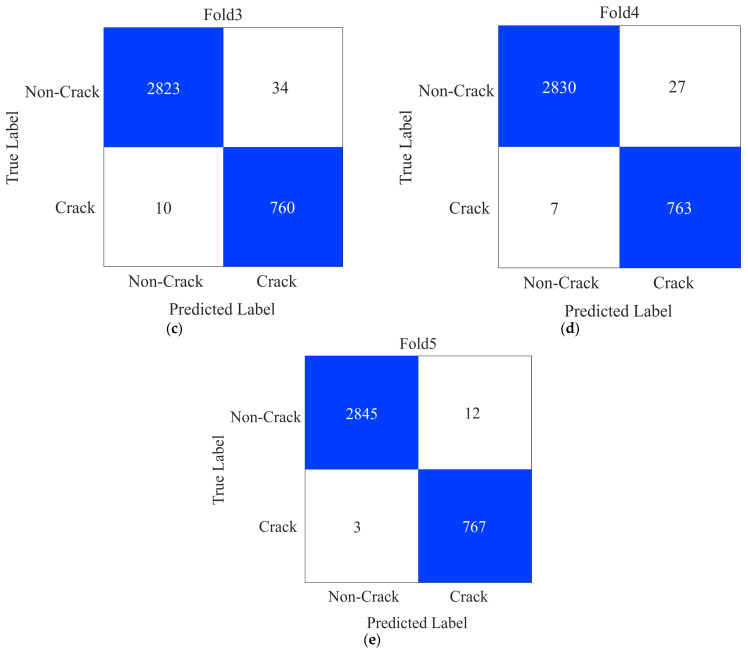
Confusion matrices of the proposed method on SDNET2018 (**a**) Fold1 (**b**) Fold2 (**c**) Fold3 (**d**) Fold4 and (**e**) Fold5.

SDNET2018 is an annotated image dataset consisting of 56,000 images. Out of these, 13,620 images are of bridge decks, 24,334 are of pavement, and 18,138 are of walls. In the proposed study, we are analyzing the building cracks. Therefore, we experimented with the images of the walls. In the wall image dataset, 3851 images are cracked, and 14,287 images are of non-crack categories. To avoid biased performance, we applied a five-fold cross-validation technique. During this process, the images were uniformly resized to dimensions of 300 × 300 pixels prior to training in a batch of 64. Furthermore, Adam’s optimization algorithm with an initial learning rate of 0.0001 was utilized to enhance the efficiency of the training. After training, we plotted confusion matrices for each fold, as depicted in Figure 6. In Figure 6, we can observe in Fold_1 that the false positive (FP) and false negative (FN) counts are 42 and 13, respectively. Notably, there is a reduction in the counts of the FP and FN values in the substituent folds.

The performance measures, precision, F1-score, kappa, recall, and accuracy, for each fold shown in Figure 5 are calculated using the formula described in the literature [40]. In Fold_1, the precision and recall values are 98.53% and 99.54%, respectively, as shown in Table 3. In the substituent fold’s performance measures, the value gradually increases. Finally, we calculated the average value of all the performance measures.

### 4.5. Performance Evaluation on the Proposed DS3

**Figure 7:**
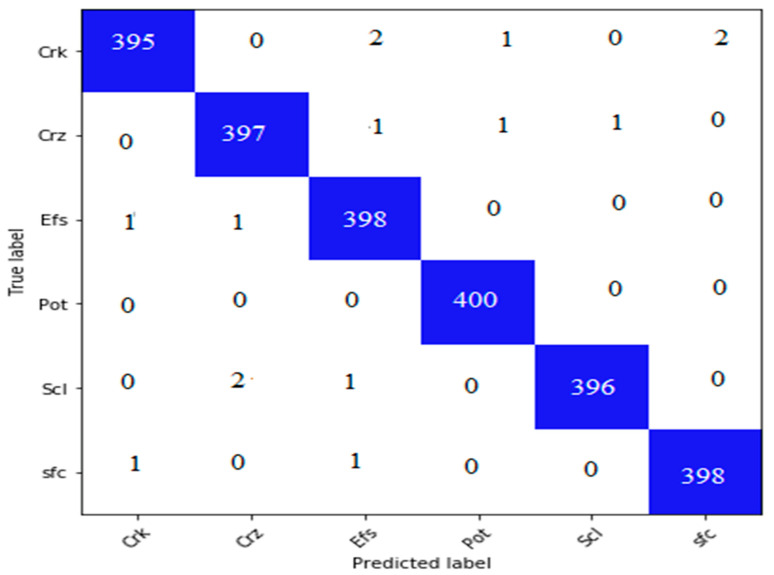
Confusion matrix on the proposed dataset.

In DS3, six types of surface cracks, Crk, Crz, Efs, Pot, Scl, and Sfc, have been experimentally evaluated using CCTNet. The DS3 dataset comprises 2115 pictures, which is insufficient for training the CCTNet model. Consequently, we implemented data augmentation techniques such as vertical, rotation, and horizontal flipping. Following augmentation, we retained a total of 2000 photos in every class. Additionally, the supplemented dataset was randomly divided into 80% for training and 20% for validation. CCTNet was trained for 200 epochs using an Adam optimizer with an initial learning rate of 0.0001. Following the model’s training, it underwent validation on the test dataset. Subsequently, a confusion matrix was constructed, shown in Figure 7. Figure 7 shows that the Crk class has the highest number of FP and FN values, whereas the lowest value can be observed in the Pot class.

We calculated several essential indicators, such as the kappa coefficient, recall, precision, F1-score, and overall accuracy, utilizing the confusion matrix shown in Figure 6. The computed indicators are shown in Table 4. Based on this assessment, it is clear that our CCTNet model has achieved remarkable performance with a kappa coefficient of 99.20%, demonstrating a nearly flawless level of concordance between the model’s predictions and the actual data. The model’s precision value of 99.33% indicates its exceptional reliability. These results highlight the precision and predictive strength of CCTNet in analyzing the DS3 dataset.

## 5. Discussion

The early detection of building cracks is crucial to mitigate potential risks to human life. Manual inspections on huge buildings are physically demanding, time consuming, and challenging. The utilization of artificial intelligence in many domains has facilitated the automation of several tasks in recent times. Several machine learning (ML) techniques have been created to detect fractures, although many rely on manually designed characteristics, leading to less-than-optimal results. On the contrary, the utilization of deep convolutional neural network (CNN) methods enables the automatic extraction of intricate information from images, leading to a notable enhancement in crack identification.

Furthermore, advancements in these approaches have been realized through data augmentation strategies, greatly enhancing their performance metrics. The corpus of existing studies on the deep CNN-based detection of building cracks is extensive. The primary focus of these studies has been on utilizing models like AlexNet, Inception V3, and VGG16 for structural cracks. Although these models are adept at classification tasks, their limited capacity for capturing global semantic features can compromise their crack detection efficacy. Additionally, the sheer volume of parameters requiring optimization in these models demands considerable computational power.

In contrast, our CCTNet framework commences with extracting spatial features using 2D CNN layers. Following this initial phase, the features are flattened to create tokens, which are relayed to the transformer module. Within this module, the network incorporates channel attention and window-based self-attention blocks, which are specifically designed to recognize and process long-range dependencies among the spatial features. The efficacy of CCTNet has been rigorously tested and validated on various datasets, including the Historical Building Crack2019, SDNET2018, and the newly introduced DS3. A comprehensive comparison of multiple deep CNN-based methodologies applied to different datasets for the detection of building cracks is presented in Table 5.

Flah et al. [41] applied a deep CNN model to classify cracks and achieved 97.00% accuracy. In similar research, Silva et al. [42] classified cracks with an accuracy of 92.27%. In another study, Yang et al. [43] utilized YOLO V3 for concrete crack detection and obtained a classification accuracy of 90%. Kumar et al. [44] utilized the sequential deep CNN model LeNet-5 for classification and achieved 98% accuracy. In the fusion-based approach, Wang et al. [45] applied Inception and ResNet-v2 to enhance the performance and obtained 96.17% classification accuracy. Chen et al. [22] classified building cracks using a deep CNN and achieved 94% accuracy. Chaiyasarn et al. [46] extracted high-dimension features using a deep CNN, and classification was performed using SVM. Özgenel et al. [47] performed a performance comparison between AlexNet and VGG16 and found that VGG16 attained the highest classification accuracy of 96%. Nugraheni et al. [48] utilized the Deca CNN to classify concrete cracks with a precision of 98.87%. Cha et al. [49] employed a deep CNN for crack identification, achieving a success rate of 98.22%. Siracusano et al. [50] utilized Bi-LSTM to classify cracks, achieving a 90% accuracy rate. Billah et al. [51] employed a CNN to accurately identify cracks of various shapes, achieving 94% accuracy. Gonzalez et al. [52] categorized deep convolutional neural networks (CNNs) for detecting cracks in buildings, achieving a precision rate of 91%. Jiang et al. [53] created an unmanned aircraft system (UAS) capable of capturing highly detailed images of cracks with a precision of 94.48%. The proposed method achieved a 98.04%, 98.59%, and 99.37% classification accuracy on the Historical Building Crack2019, SDNET2018, and the proposed DS3.

### 5.1. Training and Validation Loss

**Figure 8:**
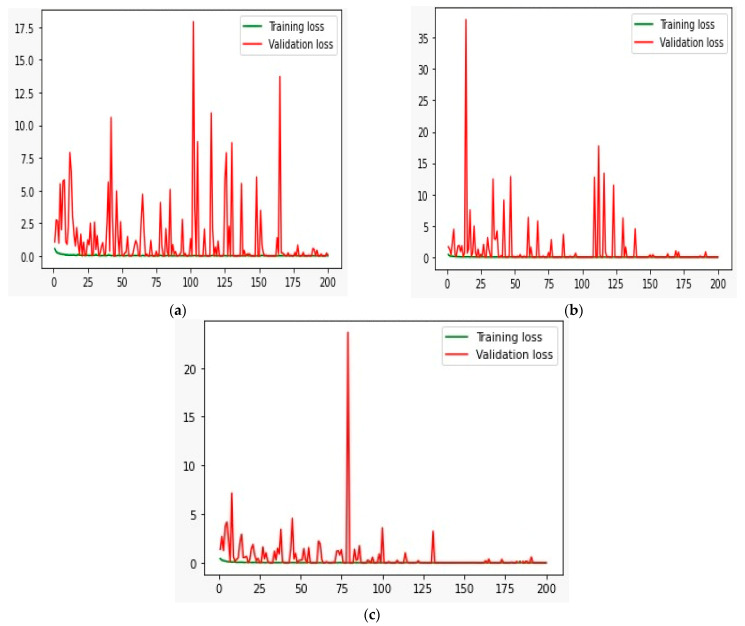
Training and validation loss of the proposed model on the Historical Building Crack2019, SDTNET2018, and the proposed DS3 shown in (**a**–**c**), respectively.

The training and validation loss provide immediate feedback on how well the model is learning. A decreasing loss over time indicates that the model is gaining the ability to generalize from the training data [54]. By comparing training loss with validation loss, it is possible to detect overfitting. Overfitting occurs when the training loss continues to decrease. At the same time, the validation loss increases, indicating that the model is learning to memorize the training data rather than generalizing from it [55]. We plotted the training (TR) and validation (VL) curve of the proposed method on the Historical Building Crack2019, SDTNET2018, and the proposed DS3, as shown in Figure 8. We can see that the model TR and VL loss on the Historical Building Crack2019 dataset has fluctuating high and low peaks. After 160 epochs, it started decreasing and reached close to zero after 190 epochs. Similarly, on the SDTNET2018 dataset, TR and VL loss was initially very high, and it started decreasing after 145 epochs. Further, it reached close to zero after 175 epochs. On the proposed DS3, the model has small peaks. After 100 epochs, TR and VL loss are much less and reach close to zero at 135 epochs.

### 5.2. Comparison with State-of-the-Art Methods

Under the same experimental conditions mentioned in Section 4.2, we evaluated the performance of VGG16 [56], InceptionV4 [57], GoogleNet [58], ResNet50 [59], AlexNet [60], DaViT [61], SI-ViT [62], and CCTNet on the Historical Building Crack2019, SDTNET2018, and our recommended DS3. VGG16 is a CNN architecture with 16 layers. The architecture employs a consistent design of 3 × 3 convolutional layers, with intermittent max pooling layers to decrease spatial dimensions. The activation function utilized throughout the network is ReLU (Rectified Linear Unit). Additionally, the convolutional layers have a fixed stride of one, and zero padding is employed to preserve the spatial resolution. The network ends with three fully connected layers, with the top two layers containing 4096 nodes each, followed by the SoftMax classifier.

InceptionV4 comprises several inception modules, which act as the essential building blocks of the design. The network collects features at various scales through parallel convolutional layers. Furthermore, the spatial dimension of the input images is reduced using a series of convolution blocks. In addition, it has kernels sizes of 1 × 1, 3 × 3, and 5 × 5 to capture the features at various scales. A global average 1 × 1 pooling layer followed by a SoftMax layer is utilized for the classification of the images.

GoogleNet outperforms its predecessors in terms of comprehensiveness, incorporating a total of 22 layers. The network employs inception modules, comprising parallel convolutional layers with varying kernel sizes and pooling layers. This design allows the network to gather features at different scales efficiently. The architecture heavily relies on 1 × 1 convolutions to decrease dimensionality and computational cost. GoogleNet replaces fully linked layers at the end of the network with global average pooling, a technique that reduces the number of parameters and addresses overfitting.

ResNet50 consists of a deep architecture including 50 layers. The skip connections enable the output of a layer to bypass one or more levels and can be reintroduced into the network at a deeper level. It facilitates the training of deep networks by enhancing the efficiency of gradient propagation during the backpropagation process. The network initiates with a convolutional layer of size 7 × 7 and a stride of two, followed by a 3 × 3 max pooling layer. Most of the network comprises four phases, each consisting of a sequence of residual blocks. A residual block comprises three convolutional layers and a skip connection. Following the residual blocks, a global average pooling layer decreases the spatial dimensions to 1 × 1. Ultimately, a wholly connected layer is utilized at the highest level to perform classification. AlexNet is a convolutional neural network with a depth of eight layers. The architecture has five convolutional layers. The initial convolutional layer comprises ninety-six filters/kernels, each with dimensions of 11 × 11 and a stride of four.

Following this layer, there is a max pooling layer and a normalizing layer. The second layer consists of 256 filters, each with a dimension of 5 × 5. They were followed by max pooling and normalization. The third layer consists of 384 filters, each with a size of 3 × 3. In addition, it is followed by two further convolutional layers that have the same architecture but do not include max pooling or normalizing. Following the convolutional layers, three completely linked layers exist. The network employs the Rectified Linear Unit (ReLU) activation function consistently. In order to address the problem of overfitting, a dropout technique is implemented prior to the initial and subsequent fully connected layers, utilizing a dropout rate of 0.5. The output layer employs a SoftMax activation function to transform the network’s output into probability scores, facilitating categorization. These models extract superficial features with many dimensions, resulting in suboptimal performance for crack identification. DaViT utilized window and channel attention to capture fine-grain spatial features. It has several variants, including small, large, and giant, whereas SI-ViT used the traditional ViT as the backbone and shuffled and un-shuffled the interconnection of spatial features in the image.

In this study, we utilized convolution and ViT networks that improve the building crack classification. The proposed CCTNet has one convolutional layer that extracts spatial features. The spatial features are flattened and passed to the ViT module. In the ViT module, MHA is achieved through CAM and W-MSA. CAM and W-MSA provide a local and global co-relation of the spatial features. Finally, classification is performed using a SoftMax activation function.

#### 5.2.1. Comparison of the Historical Building Crack2019 Dataset

VGG16, InceptionV4, GoogleNet, ResNet50, AlexNet, DaViT, SI-ViT, and CCTNet performance were evaluated under the same experimental setup on the Historical Building Crack2019 dataset. Each model underwent training using images with 300 × 300 × 3 pixels for 200 epochs. The training was performed in batches of 64, utilizing the Adam optimizer. The loss of the models was computed utilizing the binary cross-entropy function. In addition, performance metrics like kappa, recall, precision, F1-score, and accuracy are computed, as presented in Table 6. The precision values of the VGG16 model and GoogleNet are very similar, as seen in Table 6. ResNet50 achieved recall and precision values of 96.15% and 95.27%, respectively. AlexNet obtained the lowest kappa value of 88.17%. The transformer-based models, DaViT and SI-ViT, achieved better kappa values compared to classical CNN methods, whereas CCTNet achieved the highest kappa and precision values of 95.62% and 98.60%, respectively.

#### 5.2.2. Comparison of the SDTNET2018 Dataset

We performed a performance comparison of CCTNet on the SDTNET2018 dataset while maintaining the same experimental conditions. The initial images were downsized to dimensions of 300 × 300 pixels. Subsequently, they were utilized for training the model in batches of 64 for 200 epochs. The Adam optimizer was employed, utilizing an initial learning rate of 0.0001. Table 7 presents the performance metrics for each model. In Table 7, we can see that the F1-scores of VGG16 and ResNet50 are 95.55% and 95.69%, respectively. InceptionV4 and GoogleNet have identical F1-scores. DaViT and SI-ViT achieved 95.27% and 95.74% precision on the SDTNET 2019 dataset, whereas CCTNet obtained the highest precision and recall values.

#### 5.2.3. Comparison of the Proposed DS3 Dataset

The proposed DS3 dataset has six distinct types of surface cracks—Crk, Crz, Efs, Pot, Scl, and Sfc. Each class has 2000 images after data augmentation. The images were resized to 300 × 300 × 3 pixels and split into 80% and 20% for training and validation. After that, each model was trained for 200 epochs with the Adam optimizer. The performance measures are shown in Table 8. In Table 8, we can observe that VGG16 and Inception V4 have 95.78% and 95.17%, respectively. InceptionV4 and ResNet50 have a 98.04% and 98.19% classification accuracy. AlexNet achieved the lowest accuracy value of 94.29%. DaViT and SI-ViT achieved 97.94% and 98.34%, respectively, whereas CCTNet obtained the highest classification accuracy of 99.37%.

### 5.3. Precision–Recall (PR) Curve-Based Comparison

**Figure 9:**
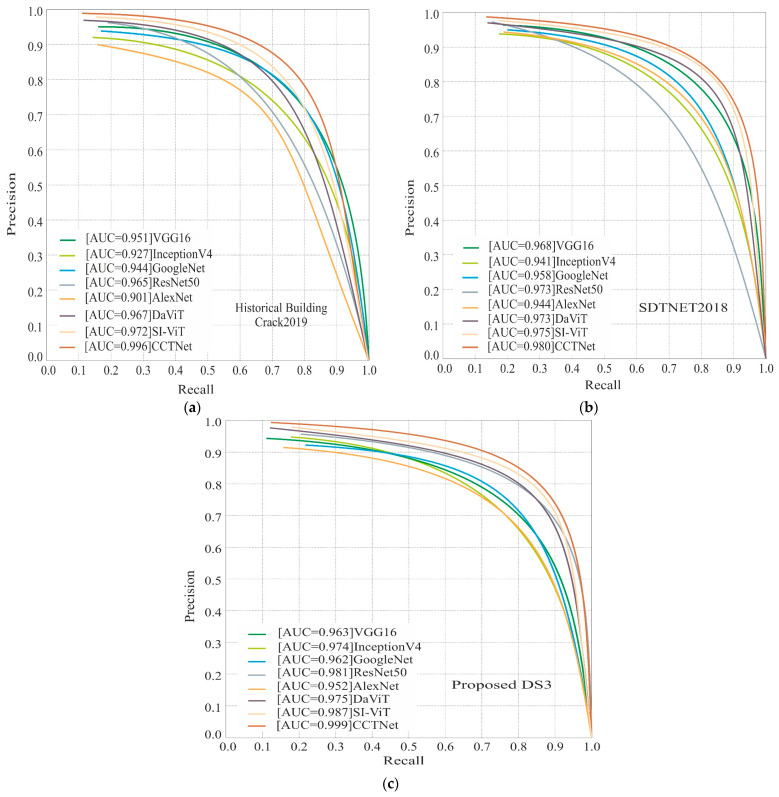
The PR curve-based comparison of the models on the (**a**) Historical Building Crack2019, (**b**) SDTNET2018, and (**c**) proposed DS3.

The PR curve is employed to assess the efficacy of a classifier, especially in scenarios where a substantial disparity in class distribution exists. This study involved assessing the model’s performance on the Historical Building Crack2019, SDTNET2018, and DS3. The Historical Building Crack2019 dataset has 757 images depicting cracks and 3139 images without any cracks. Simultaneously, SDTNET possesses 3851 images that have been successfully decrypted and 14,287 images that remain unencrypted. DS3 consists of an identical quantity of 2000 images in every class. The Historical Building Crack2019 dataset and the SDTNET2018 dataset exhibit a significant imbalance in the distribution of images between the crack and non-crack classes. Therefore, we generated a precision–recall curve, as depicted in Figure 9. In Figure 9a, the AUC (area under the curve) value of AlexNet is the smallest. The AUC values of the VGG16 and ResNet50 models exhibit minimal differences. DaViT and SI-ViT have a larger AUC value compared to traditional CNN methods. Moreover, the CCTNet that has been suggested attains the utmost worth. Figure 9b demonstrates that the AUC values of DaViT and Inception V4 have a comparable AUC value. At the same time, ResNet50 and VGG16 have AUC values that are close to each other. SI-ViT achieved a 0.975 AUC value. CCTNet obtained the highest AUC value of 0.99 on the SDTNET dataset. In Figure 9c, we can notice that the AUC values of the SI-ViT and ResNet50 are very close. The lowest value is obtained by AlexNet, whereas Inception V4 and DaViT have identical AUC values. The proposed method has a 0.99 AUC value, which is the highest in the table.

### 5.4. Attention Map-Based Comparison

**Figure 10:**
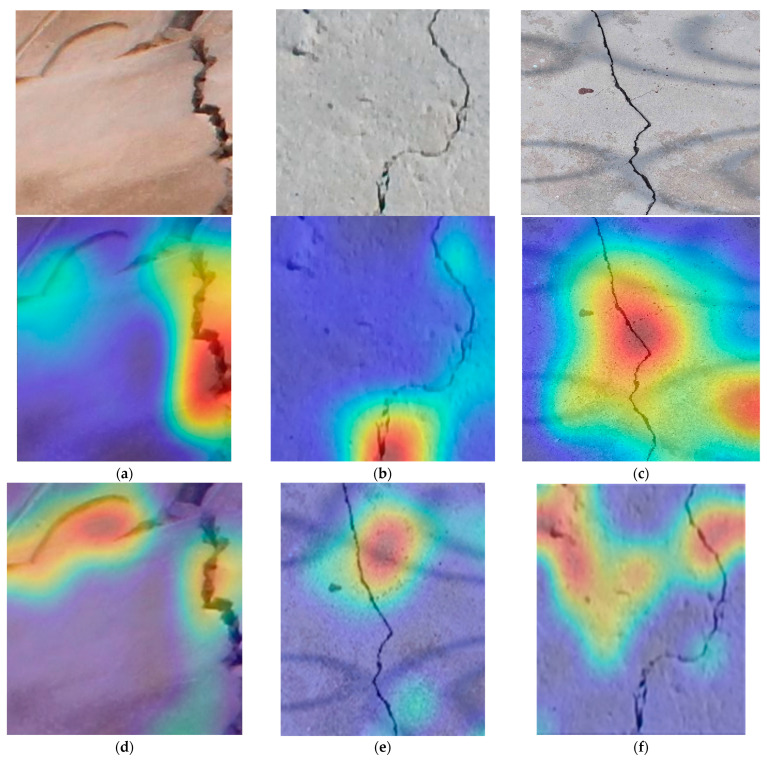

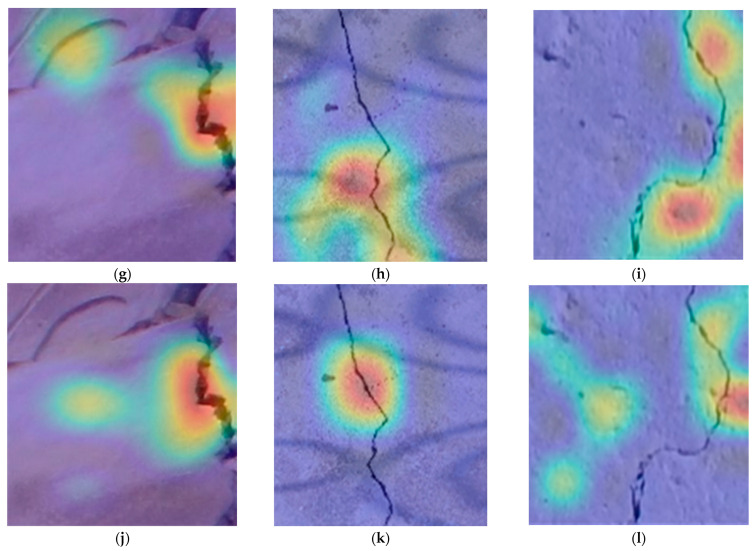
Attention map of the proposed CCTNet, ResNet50, Da-ViT, and SI-ViT on the Historical Building Crack2019, SDTNET2018, and proposed DS3 shown in (**a**–**l**), respectively. The red color indicates the highest focused region, blue is the background and whites indicate the less focused region by the model.

We plotted the attention map of CCTNet on the Historical Building Crack2019, SDTNET2018, and proposed DS3. Some of the sample images and their corresponding attention maps are shown in Figure 10. In Figure 10a, we can notice that the model has focused on the crack region to extract the features. Meanwhile, the attention map on the SDTNET2018 dataset image is high in the deep crack region. Due to the shadow in DS3, CCTNet also focused on that region, as shown in Figure 10c. Furthermore, we also plotted the attention map of ResNet50, DaViT, and SI-ViT. We can notice that ResNet50 needs to be fully capable of focusing only on the crack region. The DaViT attention map is better and focuses on the crack region, except for DS3. However, the attention map of SI-ViT is much better and focuses on the crack region.

### 5.5. Ablation Study

This section presents the ablation study of different datasets for model performance generalization.

#### 5.5.1. Performance Evaluation on the Rice Leaf Disease Dataset

**Figure 11:**
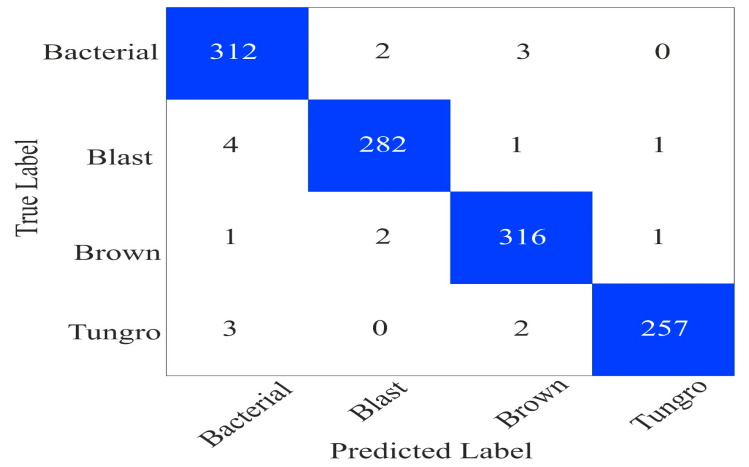
The confusion matrix on the rice leaf disease dataset.

We implemented our model on another dataset obtained from the Mendeley dataset (https://data.mendeley.com/datasets/fwcj7stb8r/1, accessed on: 5 March 2024) [63]. This dataset contains four types of diseases: Bacterial blight, Blast, Brown Spot, and Tungro in rice leaves. The images in the dataset have varying resolutions and are stored in .jpeg format. In the dataset, Bacterial blight, Blast, Brown Spot, and Tungro have 1584, 1440, 1600, and 1308 images. We randomly split 80% and 20% of the dataset for training and validation. Furthermore, we utilized the same experimental setting described in Section 4.2. The confusion matrix of the proposed model on the validation set is shown in Figure 11. The model has shown good performance and has fewer false positive and false negative values in each class.

Furthermore, we calculated the performance measures from the confusion matrix shown in Figure 11 and presented them in Table 9. In Table 9, we can notice that the model achieved 99.70% kappa and 98.29% precision values.

#### 5.5.2. Performance Evaluation on the Bridge Crack Dataset of SDTNet2018

**Figure 12:**
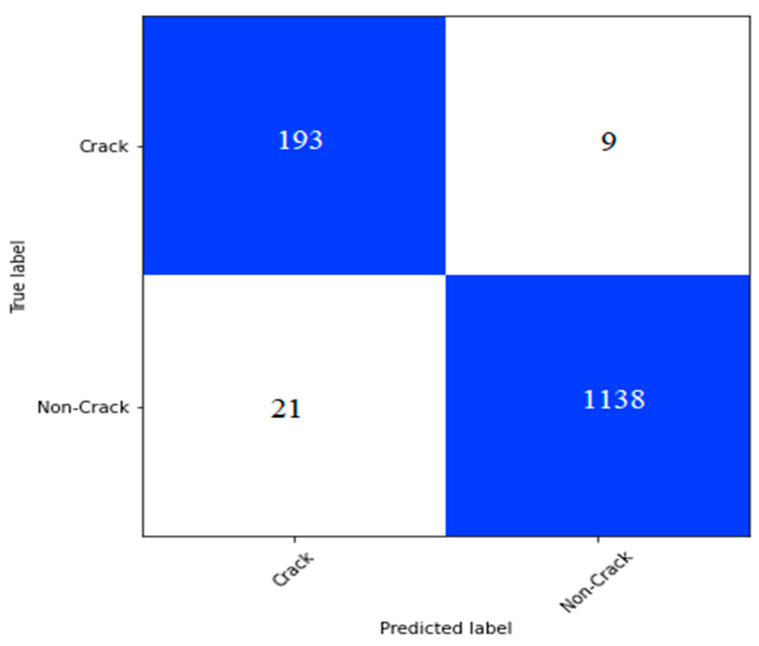
The confusion matrix on the bridge dataset of SDTNet2018.

We implemented CCTNet on the bridge crack dataset of SDTNet2018 discussed in Section 4.1. In this dataset, we have 11,595 non-crack and 2025 crack images. We split the dataset into 80%, 10%, and 10% for training, validation, and testing. After training the model, we plotted the confusion matrix on the test dataset is shown in Figure 12. In Figure 12, we can notice that the model has nine false positive and twenty-one false negative values.

From the confusion matrix shown in Figure 12, we calculated the performance measures and presented them in Table 10. We can notice that the model has obtained a 95.49% F1-score and a 94.70% recall value.

**Figure 13:**
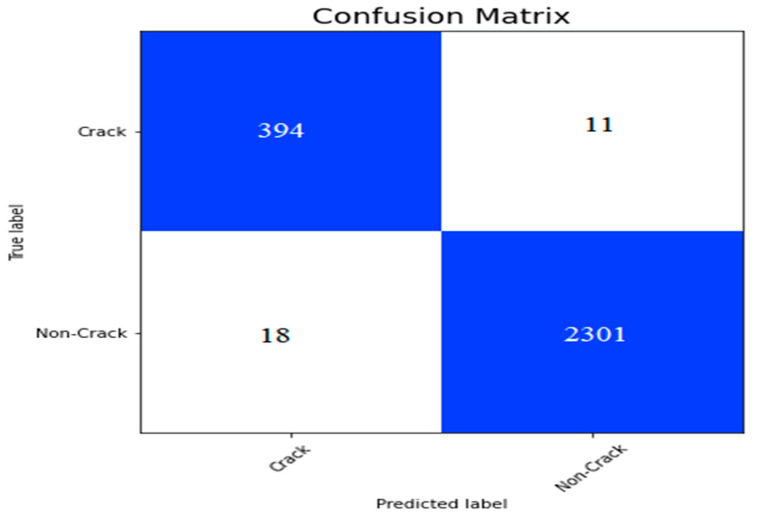
Confusion matrix for the validation set of the bridge crack dataset.

In another experiment, we split the bridge crack dataset of SDTNet into 80% and 20% for training and validation. In addition, the same experimental settings discussed in Section 4.1 were utilized for this experiment. After training the model, a confusion matrix is plotted, as shown in Figure 13. In Figure 13, we can see that 11 false positive and 18 false negative values are present.

From the confusion matrix shown in Figure 13, we calculated the performance measures presented in Table 11. In Table 11, CCTNet has achieved a 95.80% kappa value, 98.24% precision, and 98.94% classification accuracy.

## 6. Conclusions

Detecting cracks in building structures is not only a matter of preserving the integrity and value of the infrastructure but also a fundamental aspect of ensuring public safety. While various machine learning and deep learning methodologies have been explored to advance the accuracy of crack identification, traditional deep CNN approaches have limitations, most notably the substantial computational resources required due to a high number of trainable parameters and their propensity to capture high-dimensional but shallow features, which might not effectively characterize the nuanced nature of cracks. Considering these challenges, we proposed a convolution and composite attention transformer network (CCTNet). By integrating more input pixels and melding convolution channel attention with window-based self-attention mechanisms, CCTNet capitalizes on the robust feature extraction of CNNs and the extensive contextual understanding provided by self-attention mechanisms. This combined approach allows for a more detailed and comprehensive analysis of crack patterns. Empirical evaluation of the CCTNet model across the Historical Building Crack2019, SDTNET2018, and the proposed DS3 demonstrated its robust performance, achieving high precision rates of 98.60%, 98.93%, and 99.33%, respectively. CCTNet not only outperforms existing methodologies but also sets a new standard for the accurate, efficient, and reliable detection of cracks in building structures.

One of the primary limitations is the model’s generalization to varying conditions and structures not included in the training datasets. Its real-time processing capabilities are also a concern, especially when deployed on lower-end hardware. Additionally, the model’s performance is heavily reliant on access to extensive labeled data, which may not always be available. In addition, one major limitation is the selection of the patch size. During the experiment, we found that a smaller patch size reduced the training time. However, performance decreased drastically. Furthermore, a larger patch size improved the performance but at the same time, computation costs increased. After several experiments, for CCTNet, we set a patch size of 11 × 11 for all the experiments. The future scope for enhancing CCTNet includes refining its ability to generalize across diverse environmental conditions using advanced techniques, such as domain adaptation. For environmental effects, such as noise and shadow effect image preprocessing techniques, such as morphological operation and image normalization, the CNN model can be applied. In addition, optimizing the model for real-time analysis through techniques, like model pruning and hardware acceleration, is also an essential step towards practical application. Exploring semi-supervised learning could alleviate the heavy dependency on large, labeled datasets. Extending the model’s capabilities to accurately detect cracks across a variety of building materials will further enhance its utility. In addition, 

## Figures and Tables

**Table 1:** Specification of the image-capturing device for DS3.

Image-Capturing Device	Phone (Android)
Model Name	Realme 11, 5G
Subject Area	Structural health monitoring, CNN, AI
Type of Data	2D image (.jpg) format
Image Size	11,555 × 8655 pixels
Arm’s Length	26 mm
Aperture	f/1.8
Camera Rear	200 MP
Data Source Location	Shri Radha City Mathura, India
Experimental Features	Obstructions include shadows, surface debris, scaling, etc.
Exposure Period	1/40 s
Flash	None

**Table 2:** Performance measures on the Historical Building Crack2019 dataset.

Fold	Kappa (%)	Recall (%)	Precision (%)	F1-Score (%)	Accuracy (%)
Fold_1	93.64	99.35	98.09	98.72	97.95
Fold_2	94.01	99.36	98.25	98.80	98.08
Fold_3	95.62	99.68	98.57	99.12	98.59
Fold_4	96.81	99.84	98.89	99.36	98.97
Fold_5	98.00	100	99.20	99.60	99.36
Average	95.62	99.65	98.60	99.12	98.59

**Table 3:** Performance measure of CCTNet on the SDNET2018 dataset.

Fold	Kappa (%)	Recall (%)	Precision (%)	F1-Score (%)	Accuracy (%)
Fold_1	95.55	99.54	98.53	99.03	98.43
Fold_2	96.03	99.61	98.67	99.14	98.65
Fold_3	96.43	99.65	98.81	99.23	98.79
Fold_4	97.24	99.75	99.05	99.40	99.06
Fold_5	98.77	99.89	99.58	99.74	99.59
Average	96.80	99.69	98.93	99.31	98.04

**Table 4:** The performance metrics of the CCTNet model on DS3.

Kappa (%)	Recall (%)	Precision (%)	F1-Score (%)	Accuracy (%)
99.2	99.34	99.33	99.35	99.37

**Table 5:** Summary of the deep CNN and CCTNet on different datasets.

Study	Dataset	Model	Accuracy (%)
Flah et al. [41]	40,000 images	DCNN	97.00
Silva et al. [42]	3500 images	DCNN	92.27
Yang et al. [43]	1600 images	YOLO V3	90.00
Kumar et al. [44]	2068 images	LeNet-5	98.00
Wang et al. [45]	3000 images	Inception-ResNet-v2	96.17
Chen et al. [22]	3600 images	DCNN	94.00
Chaiyasarn et al. [46]	6002 images	DCNN and SVM	82.94
Özgenel et al. [47]	40,000 images	VGG16	96.00
Nugraheni et al. [48]	10,000 images	DCL-NN	98.87
Cha et al. [49]	40,000 images	DCNN	98.22
Siracusano et al. [50]	15,000 images	Bi-LSTM	90.00
Billah et al. [51]	21,996 images	CNN	94.00
Gonzalez et al. [52]	10,000 images	DCNN	91.00
Jiang et al. [53]	1330 images	UAS	94.48
Proposed method	18,138 images	CNN + ViT	98.04
	4643 images	CNN + ViT	98.59
	12,000 images	CNN + ViT	99.37

**Table 6:** Performance evaluation of different methods on the Historical Building Crack2019.

Method	Kappa (%)	Recall (%)	Precision (%)	F1-Score (%)	Accuracy (%)
VGG16 [56]	90.16	93.85	95.24	94.53	93.52
InceptionV4 [57]	89.74	94.82	91.67	96.11	92.35
GoogleNet [58]	90.48	92.64	95.92	94.25	94.28
ResNet50 [59]	93.56	95.27	96.15	95.70	95.43
AlexNet [60]	88.17	91.05	92.37	91.70	93.18
DaViT [61]	93.36	95.67	94.23	94.93	95.07
SI-ViT [62]	94.92	96.51	95.29	95.89	97.13
CCTNet	95.62	99.65	98.60	99.12	98.59

**Table 7:** Performance comparison of the SDTNET2019 dataset.

Method	Kappa (%)	Recall (%)	Precision (%)	F1-Score (%)	Accuracy (%)
VGG16 [56]	92.43	94.36	96.78	95.55	95.87
InceptionV4 [57]	93.18	95.27	93.32	94.28	95.19
GoogleNet [58]	91.89	93.83	94.81	94.13	94.89
ResNet50 [59]	94.76	96.15	95.25	95.69	96.36
AlexNet [60]	90.58	92.79	93.78	93.28	93.49
DaViT [61]	94.87	96.12	95.27	95.69	96.37
SI-ViT [62]	95.13	98.02	95.74	96.86	98.18
CCTNet	97.80	99.69	98.93	99.31	99.24

**Table 8:** Performance comparison of the proposed DS3.

Method	Kappa (%)	Recall (%)	Precision (%)	F1-Score (%)	Accuracy (%)
VGG16 [56]	94.35	96.84	95.78	96.30	97.56
InceptionV4 [57]	95.12	97.47	95.17	96.31	98.04
GoogleNet [58]	93.82	95.35	94.59	94.96	95.29
ResNet50 [59]	96.13	97.85	98.23	98.03	98.19
AlexNet [60]	92.27	94.37	93.76	84.06	94.29
DaViT [61]	97.30	96.89	97.68	97.28	97.94
SI-ViT [62]	98.15	97.29	98.46	97.87	98.34
CCTNet	99.2	99.34	99.33	99.35	99.37

**Table 9:** Performance evaluation on the rice leaf disease dataset.

Kappa (%)	Recall (%)	Precision (%)	F1-Score (%)	Accuracy (%)
97.70	98.37	98.29	98.33	98.35

**Table 10:** Performance evaluation on the unseen dataset of SDTNet2018.

Kappa (%)	Recall (%)	Precision (%)	F1-Score (%)	Accuracy (%)
91.50	94.70	96.86	95.49	97.79

**Table 11:** Performance evaluation on the validation dataset of SDTNet2018.

Kappa (%)	Recall (%)	Precision (%)	F1-Score (%)	Accuracy (%)
95.80	97.58	98.24	97.91	98.94

## Data Availability

The data used in the study can be downloaded from https://data.mendeley.com/datasets/xfk99kpmj9/1 (accessed on 8 June 2024) and https://digitalcommons.usu.edu/all_datasets/48/ (accessed on 8 June 2024).
